# [Retracted] KIFC1 promotes the proliferation of hepatocellular carcinoma *in vitro* and *in vivo*

**DOI:** 10.3892/ol.2023.13931

**Published:** 2023-06-26

**Authors:** Xing Wang, Meng Wang, Xing-Yue Li, Jian Li, Dian-Peng Zhao

Oncol Lett 18: 5739–5746, 2019; DOI: 10.3892/ol.2019.10985

Following the publication of this paper, it was drawn to the Editor's attention by a concerned reader that certain of the western blotting data shown in Fig. 3D on p. 5744 and immunohistochemical data shown in Fig. 4C on p. 5745 were strikingly similar to data that had appeared in different form in different articles published by different authors at different research institutes.

Owing to the fact that some of the data in the above article had already been published, or were under consideration for publication, prior to its submission to *Oncology Letters*, the Editor has decided that this paper should be retracted from the Journal. The authors were asked for an explanation to account for these concerns, but the Editorial Office did not receive any reply. The Editor apologizes to the readership for any inconvenience caused.

